# Supplementary material for: Transcription of MERVL retrotransposons is required for preimplantation embryo development
Source: Nat Genet. 2023 Mar 2;55(3):484–95. doi: 10.1038/s41588-023-01324-y (PMC10011141; doi:10.1038/s41588-023-01324-y)
Supplement: Supplementary file 1 — Reporting Summary [file 41588_2023_1324_MOESM1_ESM.pdf]

nature portfolio

Corresponding author(s): Haruhiko Siomi

Last updated by author(s): Sep 2, 2022

Reporting Summary

Nature Portfolio wishes to improve the reproducibility of the work that we publish. This form provides structure for consistency and transparency in reporting. For further information on Nature Portfolio policies, see our Editorial Policies and the Editorial Policy Checklist.

Statistics

For all statistical analyses, confirm that the following items are present in the figure legend, table legend, main text, or Methods section.

n/a	Confirmed	
☐	☒	The exact sample size (*n*) for each experimental group/condition, given as a discrete number and unit of measurement
☐	☒	A statement on whether measurements were taken from distinct samples or whether the same sample was measured repeatedly
☐	☒	The statistical test(s) used AND whether they are one- or two-sided<br>*Only common tests should be described solely by name; describe more complex techniques in the Methods section.*
☒	☐	A description of all covariates tested
☐	☒	A description of any assumptions or corrections, such as tests of normality and adjustment for multiple comparisons
☐	☒	A full description of the statistical parameters including central tendency (e.g. means) or other basic estimates (e.g. regression coefficient) AND variation (e.g. standard deviation) or associated estimates of uncertainty (e.g. confidence intervals)
☐	☒	For null hypothesis testing, the test statistic (e.g. *F*, *t*, *r*) with confidence intervals, effect sizes, degrees of freedom and *P* value noted<br>*Give P values as exact values whenever suitable.*
☒	☐	For Bayesian analysis, information on the choice of priors and Markov chain Monte Carlo settings
☒	☐	For hierarchical and complex designs, identification of the appropriate level for tests and full reporting of outcomes
☐	☒	Estimates of effect sizes (e.g. Cohen's *d*, Pearson's *r*), indicating how they were calculated

Our web collection on statistics for biologists contains articles on many of the points above.

Software and code

Policy information about availability of computer code

Data collection	Data were collected with GNU Wget, 64-bit miniconda3 package 'conda install' and SRA Toolkit 'prefetch' from the following web databases.
Data analysis	Source code for all software and tools used in this study with documentation, examples and additional information, is available at following URLs:<br>https://github.com/GenomeImmunobiology/Sakashita_et_al_2020 (best-match TE annotation set)<br>https://dfam.org/family/DF0003918/summary (MERVL sequence)<br>https://www.biosearchtech.com/support/tools/design-software/stellaris-probe-designer (Stellaris Probe Designer for smFISH)<br>https://bedtools.readthedocs.io/en/latest (bedtools v2.30.0)<br>https://www.ncbi.nlm.nih.gov/books/NBK279690 (BLAST v2.6.0+)<br>https://www.ncbi.nlm.nih.gov/geo/query/acc.cgi?acc=GSE45719 (scRNA-seq dataset in mouse preimplantation embryos)<br>https://github.com/alexdobin/STAR (STAR RNA-seq aligner v2.5.3a)<br>http://hgdownload.cse.ucsc.edu/goldenpath/mm10/bigZips/mm10.fa.gz (GRCm38/mm10)<br>https://ftp.ebi.ac.uk/pub/databases/gencode/Gencode_mouse/release_M25/gencode.vM25.annotation.gtf.gz (GENCODE gene annotation)<br>http://subread.sourceforge.net (Subread v2.0.1)<br>https://bioconductor.org/packages/release/bioc/html/DESeq2.html (DESeq2 v1.16.1)<br>https://maayanlab.cloud/Enrichr (Enrichr)<br>https://dbtmee.hgc.jp (DBTMEE v2, Database of Transcriptome in Mouse Early Embryos)<br>https://software.broadinstitute.org/morpheus (Morpheus)<br>https://software.broadinstitute.org/software/igv/igvtools (IGVTools v2.9.2)<br>https://www.bioinformatics.babraham.ac.uk/projects/seqmonk (SeqMonk v1.48.0)<br>http://bowtie-bio.sourceforge.net/bowtie2 (bowtie2 v2.4.4)<br>https://hbctraining.github.io/Intro-to-ChIPseq/lessons/05_peak_calling_macs.html (MACS2 v2.1.4)<br>https://deeptools.readthedocs.io/en/develop (deepTools v3.1.3)

https://bioconductor.org/packages/release/bioc/html/groHMM.html (groHMM v1.24.0)
https://github.com/shenlab-sinai/ngsplot (ngsplot v2.4.4)
http://homer.ucsd.edu/homer (HOMER v4.9)
https://systems.crump.ucla.edu/hypergeometric (Hypergeometric p-value calculator)
https://www.socscistatistics.com/tests/chisquare (Chi Square Calculator)
http://great.stanford.edu/public/html (GREAT v4.0.4)
https://imagej.net/Fiji/Downloads (Fiji — ImageJ)
https://www.bioinformatics.babraham.ac.uk/projects/trim_galore (TrimGalore v0.6.4)

For manuscripts utilizing custom algorithms or software that are central to the research but not yet described in published literature, software must be made available to editors and reviewers. We strongly encourage code deposition in a community repository (e.g. GitHub). See the Nature Portfolio guidelines for submitting code & software for further information.

Data

Policy information about availability of data

All manuscripts must include a data availability statement. This statement should provide the following information, where applicable:

- Accession codes, unique identifiers, or web links for publicly available datasets
- A description of any restrictions on data availability
- For clinical datasets or third party data, please ensure that the statement adheres to our policy

The total RNA-seq and miniATAC-seq data reported in this study are deposited in the Gene Expression Omnibus (GEO) under accession code GSE196520. Other scRNA-seq dataset from GSE45719 are described and cited in the manuscript.

Field-specific reporting

Please select the one below that is the best fit for your research. If you are not sure, read the appropriate sections before making your selection.

☒ Life sciences ☐ Behavioural & social sciences ☐ Ecological, evolutionary & environmental sciences

For a reference copy of the document with all sections, see nature.com/documents/nr-reporting-summary-flat.pdf

Life sciences study design

All studies must disclose on these points even when the disclosure is negative.

Sample size	No statistical methods were used to predetermine sample sizes. Sample sizes for smFISH, IF, RT-qPCR, WB, developmental monitoring of embryos, EU incorporation assay, total RNA-seq and miniATAC-seq are consistent with current standards.
Data exclusions	No data were excluded from analyses.
Replication	We confirmed consistent results between three-to-six independent biological replicates (and/or experiments) for qPCR, smFISH and IF and EU incorporation assay. For Nextgen sequencing analysis, we also confirmed consistent results between two independent biological replicates for total RNA-seq and miniATAC-seq experiments based on Pearson's correlation coefficient of gene expressions and read enrichments, as determined by SeqMonk. The number of replicates/experiments has been described in the figures, legends and main text.
Randomization	The experiments were not randomized. Sample were allocated as either control or experimental (KD, CRISPRi and/or rescue) groups.
Blinding	The experiments were not blinding. However our analytical pipeline for each experiment followed uniform criteria applied to all samples, allowing us to analyze data, unbiasedly.

Reporting for specific materials, systems and methods

We require information from authors about some types of materials, experimental systems and methods used in many studies. Here, indicate whether each material, system or method listed is relevant to your study. If you are not sure if a list item applies to your research, read the appropriate section before selecting a response.

Materials & experimental systems

n/a	Involved in the study	
☐	☒	Antibodies
☐	☒	Eukaryotic cell lines
☒	☐	Palaeontology and archaeology
☐	☒	Animals and other organisms
☒	☐	Human research participants
☒	☐	Clinical data
☒	☐	Dual use research of concern

Methods

n/a	Involved in the study	
☒	☐	ChIP-seq
☒	☐	Flow cytometry
☒	☐	MRI-based neuroimaging

Antibodies

Antibodies used	The methods section of the manuscript contains information on all antibodies.<br>Mouse anti-MERVL Gag monoclonal antibody (A2 and C1, 1/5 dilution, generated in our lab (Guo Y., in prep))<br>Mouse anti-β-tubulin monoclonal antibody (E7, 1/2000 dilution, E7, Developmental Studies Hybridoma Bank)<br>Rabbit anti-OCT4 monoclonal antibody (EPR17929, 1/100 dilution, ab181557, abcam)<br>Mouse anti-CDX2 monoclonal antibody (CDX2-88, 1/100 dilution, ab157524, abcam)<br>Mouse anti-E-Cadherin monoclonal antibody (36, 1/100 dilution, 610182, BD Transduction Laboratories)<br>Rabbit anti-Cleaved Caspase-3 polyclonal antibodies (1/200, 9661, CST)<br>Rabbit anti-Phospho-S15-p53 polyclonal antibodies (1/200 dilution, 9284, CST)<br>HRP-conjugated goat anti-mouse IgG secondary antibody (1/10000 dilution, #330, MBL life science)<br>Alexa Fluor 488-conjugated goat anti-mouse IgG secondary antibody (1/500 dilution, A-11001, Thermo Fisher Scientific)<br>Alexa Fluor 568-conjugated goat anti-mouse IgG secondary antibody (1/500 dilution, A-11004, Thermo Fisher Scientific)<br>Alexa Fluor 488-conjugated goat anti-rabbit IgG secondary antibody (1/500 dilution, A-11008, Thermo Fisher Scientific)<br>Alexa Fluor 568-conjugated goat anti-rabbit IgG secondary antibody (1/500 dilution, A-11011, Thermo Fisher Scientific)
Validation	Antibodies used for immunofluorescence analysis and western blotting were validated by manufacturers (or us).<br>Mouse anti-MERVL Gag monoclonal antibody : in this study and Guo Y., in prep<br>Mouse anti-β-tubulin monoclonal antibody : https://dshb.biology.uiowa.edu/E7_2<br>Rabbit anti-OCT4 monoclonal antibody : https://www.abcam.co.jp/oct4-antibody-epr17929-chip-grade-ab181557.html<br>Mouse anti-CDX2 monoclonal antibody : https://www.abcam.co.jp/cdx2-antibody-cdx2-88-ab157524.html<br>Mouse anti-E-Cadherin monoclonal antibody : https://www.citeab.com/antibodies/2412208-610182-bd-transduction-laboratories-purified-mouse<br>Rabbit anti-Cleaved Caspase-3 polyclonal antibodies : https://www.cellsignal.jp/products/primary-antibodies/cleaved-caspase-3-asp175-antibody/9661<br>Rabbit anti-Phospho-S15-p53 polyclonal antibodies : https://www.cellsignal.jp/products/primary-antibodies/phospho-p53-ser15-antibody/9284 and Cui W. et al. Sci Rep., 2016 : https://www.nature.com/articles/srep37396<br>HRP-conjugated goat anti-mouse IgG secondary antibody : https://ruo.mbl.co.jp/bio/dtl/dtlfiles/330_v5.pdf<br>Alexa Fluor 488-conjugated goat anti-mouse IgG secondary antibody : https://www.thermofisher.com/antibody/product/Goat-anti-Mouse-IgG-H-L-Cross-Adsorbed-Secondary-Antibody-Polyclonal/A-11001<br>Alexa Fluor 568-conjugated goat anti-mouse IgG secondary antibody : https://www.thermofisher.com/antibody/product/Goat-anti-Mouse-IgG-H-L-Cross-Adsorbed-Secondary-Antibody-Polyclonal/A-11004<br>Alexa Fluor 488-conjugated goat anti-rabbit IgG secondary antibody : https://www.thermofisher.com/antibody/product/Goat-anti-Rabbit-IgG-H-L-Cross-Adsorbed-Secondary-Antibody-Polyclonal/A-11008<br>Alexa Fluor 568-conjugated goat anti-rabbit IgG secondary antibody : https://www.thermofisher.com/antibody/product/Goat-anti-Rabbit-IgG-H-L-Cross-Adsorbed-Secondary-Antibody-Polyclonal/A-11011

Eukaryotic cell lines

Policy information about cell lines

Cell line source(s)	Doxycycline (Dox)-inducible Dux ESC line, generated in our laboratory (Li T.D., et al. 2022) was used in this study.
Authentication	Since these cells were easily distinguished based on colony morphologies, cell lines have been authenticated by microscopic inspection.
Mycoplasma contamination	None of the cell lines used have been tested.
Commonly misidentified lines<br>(See ICLAC register)	No commonly misidentified cell lines were used in this study.

Animals and other organisms

Policy information about studies involving animals; ARRIVE guidelines recommended for reporting animal research

Laboratory animals	Wild-type BDF1 female and male mice at 56-84 days of age were used for the knockdown (KD) experiment. Mice were maintained and fed ad libitum with standard diet and water in a temperature-, humidity-, light-controlled room (23±3 degree Celsius, humidity of 50±10%, 14 light/10 dark cycle).
Wild animals	No wild animals were used in this study.
Field-collected samples	No field-collected samples were used in this study.
Ethics oversight	Mice were maintained and used according to the guidelines of the Institutional Animal Care and Use Committee (protocol no. 09105-(10) and 11045-(6)) at Keio University.

Note that full information on the approval of the study protocol must also be provided in the manuscript.
