# Supplementary figures and images for: Transcription of MERVL retrotransposons is required for preimplantation embryo development
Source: Nat Genet. 2023 Mar 2;55(3):484–95. doi: 10.1038/s41588-023-01324-y (PMC10011141; doi:10.1038/s41588-023-01324-y)

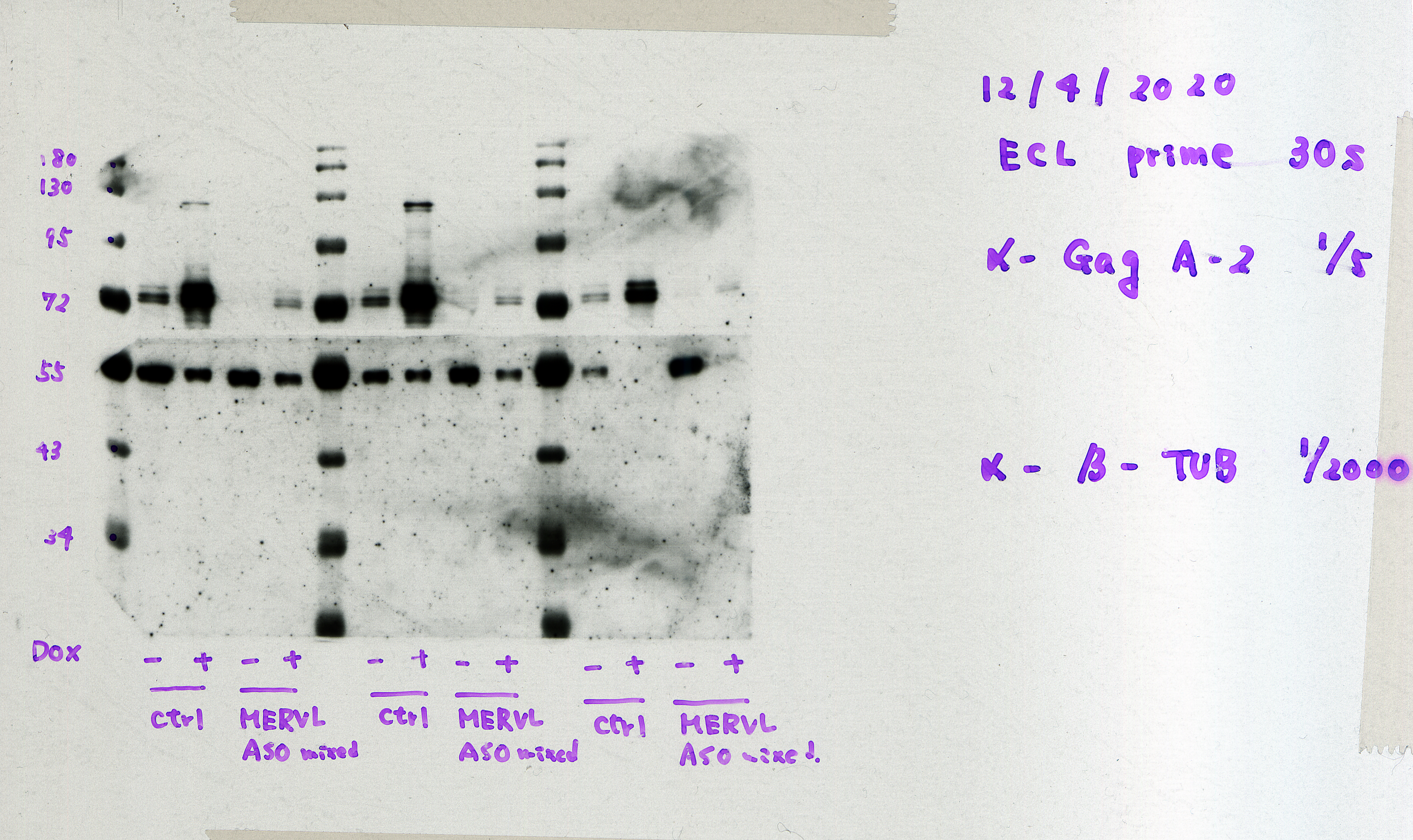

Supplement: Source Data Extended Data Fig. 1h — Unprocessed western Blots [file 41588_2023_1324_MOESM18_ESM.tif]
